# International Hahnemann Association

**Published:** 1881-02

**Authors:** 


### THE INTERNATIONAL HAHNEMANN ASSOCIATION.

Bureau of Materia Medica of the International Hahnemann Association.

Ad. Lippe, M. D., (Philadelphia) Chairman, H. C. Allen, M. D., (Ann Arbor), T. F. Pomeroy, M. D., (Jersey City), C. Pearson, M. D., (Washington, D. C.)

The Chairman has appointed the following physicians on the bureau of CLINICAL MEDICINE of the International Hahnemann Association for the meeting to be held in June 1881. Each member is at liberty to select, and write, upon any medical topic, pertaining to this bureau he may desire.

Wm. P. Wessselhoeft, M. D., Boston, Saml. Swan, M, D., New York, Geo. F. Foote, M. D., Stamford, Conn.; L. Kenyon, M. D., Buffalo, New York; E. W. Berridge, M. D., London, J. P. Mills, M. D., Chicago; Edward Rushmore, M. D. Plainfield, N. J.; C. Pearson, M. D., Washington, D. C.; Chairman.

